# Comparison of Rat Connective Tissue Response to BioMTA, Angelus MTA, and Root MTA

**DOI:** 10.1155/2021/7415302

**Published:** 2021-08-31

**Authors:** Marzieh Alimohammadi, Sina Mirzaee-Rad, Farideh Feizi, Hadi Shirzad Juybari, Akam Saeidi, Hemmat Gholinia

**Affiliations:** ^1^Department of Oral Medicine, Dental Faculty, Shahid Beheshti University of Medical Sciences, Tehran, Iran; ^2^Dental Materials Research Center, Health Research Institute, Babol University of Medical Sciences, Babol, Iran; ^3^Cellular and Molecular Biology Research Center, Health Research Institute, Babol University of Medical Sciences, Babol, Iran; ^4^Department of Orthodontics, School of Dentistry, Babol University of Medical Sciences, Babol, Iran; ^5^Oral Health Research Center, Health Research Institute, Babol University of Medical Sciences, Babol, Iran; ^6^Master of Science in Statistics, Health Research Institute, Babol University of Medical Sciences, Babol, Iran

## Abstract

Due to the widespread use of MTA in dentistry and various brands of this product, we decided to compare the three brands available in the country market by their biocompatibility. We divided 20 male Wistar rats into four groups. After local anesthesia and washing, we made two incisions on both sides (4 incisions in total). The experimental groups were Angelus MTA (Angelus, Brazil), BioMTA (CERKAMED, Poland), Root MTA (Dr. Lotfi, Tabriz, Iran), and the control group. The resulting paste was placed in a tube and implanted subcutaneously into male Wistar rats. Wistar rats were sacrificed 7, 15, 30, and 60 days later, with high anesthetic doses. The sample implanted in 10% formalin was stabilized after tissue processing and H&E staining under a microscope. The inflammatory reaction in the tissues received different scores at the beginning of the tube opening. BioMTA had the highest inflammatory response among the groups, but the difference was not statistically significant (*p* > 0.05). Also, there was no significant difference between the groups' granulation and calcification (*p* < 0.05). There was a significant difference between BioMTA, Angelus MTA, Root MTA, and control groups in fibrous capsule formation (*p* < 0.05). Angelus MTA showed the lowest mean fibrous capsule formation in all periods. The effects of Angelus MTA, Root MTA, and BioMTA on connective tissue were investigated and compared. According to this study, these materials have good biocompatibility. According to the findings and statistical analysis, Angelus MTA has the most biocompatibility.

## 1. Introduction

The primary goal of endodontics procedures is to clear and fill the root canal by creating a hermetic seal in the apex of the root [1], which is carried out to prevent reinfection and to repair root periapical tissue [2]. The materials used in endodontics, especially those used as root-end fillers, directly contact soft and hard periodontal tissues. Therefore, it is essential to adapt nontoxic and high-compatible substances [3]. Mineral Trioxide Aggregate (MTA) is a root-end filler material that was first developed by Torabinejad et al. in 1995 at the University of Loma Linda [4]. MTA with properties such as pH = 12.5, long hardening time, high seal strength, bacterial leakage prevention, and tissue compatibility is known as a suitable material for root-end filling. This material is easy to use and stimulates the formation of bone and dentin [5]. It was approved and commercialized by the FDA as ProRoot MTA. Until 2006, two commercial models of MTA were available, including ProRoot MTA and Angelus MTA [6]. The main constituents of MTA are cement (75%), bismuth oxide (20%), and gypsum (5%) and a few amount of MgO, K2sou, and Na2sou. Cement is a combination of dicalcium silicate, tricalcium silicate, tricalcium aluminate, and tetracalcium aluminoferrite [7]. Research has shown that MTA has antimicrobial activity [8] and is biocompatible in recovering periapical lesions [9, 10]. MTA induces the repair of periradicular hard and soft tissues [11]. It plays an essential role in optimal tissue reactions without multiple inflammatory responses, the formation of fibrous capsules, and induction of mineralized repair tissue formation [12]. It can also be used selectively for various clinical applications such as pulp coating, sealing and repair of root perforations, and apical barriers in open apexes. Biocompatibility of dental materials can be tested using in vitro cytotoxicity tests, subcutaneous tissue response, and bone implants [1]. Due to the importance of biocompatibility of materials used in dentistry and the lack of a similar study, we decided to compare the biocompatibility of domestic samples including Root MTA, BioMTA, and Angelus MTA. BioMTA (CERKAMED, Poland) has recently been introduced to the Iranian market, which has a lower setting time, maximum plasticity, three times higher hardness, and complete biocompatibility. It consists of hydroxyapatite and nanoparticles in its structure [13]. Due to the importance of biocompatibility of materials used in dentistry and the lack of a similar study, we decided to compare the biocompatibility of domestic samples including Root MTA, BioMTA, and Angelus MTA. BioMTA (CERKAMED, Poland) has recently been introduced to the Iranian market, which has a lower setting time, maximum plasticity, three times higher hardness, and complete biocompatibility. It includes hydroxyapatite and nanoparticles in its structure. Angelus, Brazil, has recently introduced a new product called Bio with new features, including reduced setting time, low solubility, bactericidal, and optimal tissue compatibility [14]. On the other hand, various domestically made MTAs, e.g., Lotfi Company (Root MTA, Tabriz, Iran), are affordable and always attract dentists' attention. This study aimed to compare rat connective tissue response to BioMTA, Angelus MTA, and Root MTA.

## 2. Methods and Materials

This study was approved by the Research Council of Babol University of Medical Sciences (Ethics Committee IR.MUBABOL.HRI.REC.1398.057). The present work was performed in the laboratory animal research center of Babol University of Medical Sciences and the histology laboratory of Babol University of Medical Sciences under animal care principles in the animal laboratory. 20 healthy adult white male Wistar rats weighing approximately 200 to 250 g were divided into 4 groups (*n* = 5). Rats had not been experimentally tested before, and their dorsal subcutaneous tissue was normal. Rats were anesthetized by intramuscular injection of ketamine (60 mg/kg) and xylazine (10 mg/kg). The back hairs of rats were shaved and disinfected with 5% iodine solution. Small longitudinal incisions approximately 0.5 cm in length were made on the dorsal surface of all rats. These incisions were made at equal intervals (3 cm) on both sides of the upper part of the dorsal surface and the 2 sides of the lower part of the dorsal surface so that we had a total of 4 incisions on the dorsal surface of each rat.

The experimental groups were divided into 4 groups including (1) Angelus MTA (Angelus, Brazil), (2) BioMTA (CERKAMED, Poland), (3) Root MTA (Dr. Lotfi, Tabriz, Iran), and (4) the control group. Sterile polyethylene tubes with a diameter of 1.1 mm and a length of 0.5 cm were filled by 3 groups of MTA paste, which was prepared by combining 3 mg of powder with 1 ml of distilled water solution. The empty tube was used as a negative control. These tubes were then coded and administered into incisions in the subcutaneous tissue of the rats (2 tubes on one side and 2 tubes on the other side, 3 cm apart). In each of these sections, a polyethylene tube containing 3 types of MTA and a control group with specific coding was placed and each rat was administered by 4 experimental groups. After the tubes were placed, the wounds were sutured. The rats were then sacrificed on days 7, 15, 30, and 60, respectively, using high anesthetic doses. After touching the skin on the back of the rats in the previous incision region and finding the exact location of the tube, a longitudinal incision was made. Then, the samples were fixed for histological examination in 10% formalin buffer solution with pH = 7 for 48 hours. After fixing the isolated tissue samples in 10% formalin, sections with a thickness of 3µm were taken and then stained with hematoxylin-eosin. Slides were evaluated under the OLYMPUS BX41TF microscope, Tokyo, Japan, with a magnification of 100*x*. Fibrous capsules were measured by Motic Image Plus 2.0 ML software (MICRO-OPTIC INDUSTRIAL GROUP) differently, and their mean was taken. Fibrous capsules were classified as “thin” or “thick,” with <150 *μ*m or> 150 *μ*m thickness. The sections were examined further for the presence of granular tissue, inflammatory response, calcification, and foreign body giant cell. Each slide was examined by an expert histologist under an OLYMPUS BX41TF (Tokyo, Japan) light microscope with a magnification of ×400 to evaluate inflammation scores. Similar to the study by Hoshyari et al., the rate of inflammation was scored based on the number of inflammatory cells. Score1: no inflammatory cells are seen under a light microscope or few are seen (no reaction), score2: less than 25 inflammatory cells are seen under a light microscope (mild reaction), score3: 25 to 125 cell inflammation can be seen under a light microscope (moderate reaction), and score4: 125 or more inflammatory cells can be seen under a light microscope (severe reaction) [15]. The result scores were entered into SPSS software version 22 ANOVA, and Tukey post hoc tests were used if there was data normality, and Kruskal–Wallis and Mann–Whitney tests were used if there was no data normality. *p* < 0.05 was considered significant.

## 3. Results

**Figure 1:**
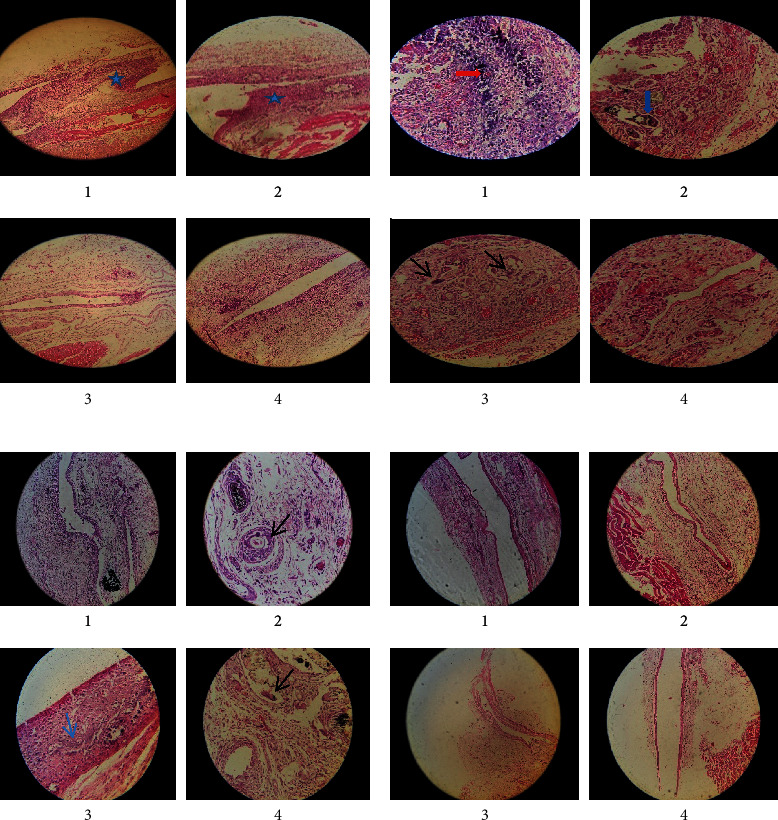
(a) Sample tissue of microtube implantation site on the 7^th^ day with hematoxylin-eosin staining; 1- MTA Bio, 2- MTA Root, 3- MTA Angelus, and 4- MTA control; magnification ×10. (b) Sample tissue of the microtube implantation site in the 7^th^ day with hematoxylin-eosin staining. Black spike: giant cell, blue spike: foregin body, and red spike: calcification; 1-MTA Bio, 2- MTA Root, 3- MTA Angelus, and 4- MTA control; magnification: ×40. (c) Sample tissue of the microtube implantation site on the 15^th^ day with hematoxylin-eosin staining; magnification ×10, ×40; black spike: giant cell and blue spike: calcification. (d) Sample tissue of the microtube implantation site on the 60th day with hematoxylin-eosin staining; magnification ×10.

**Figure 2:**
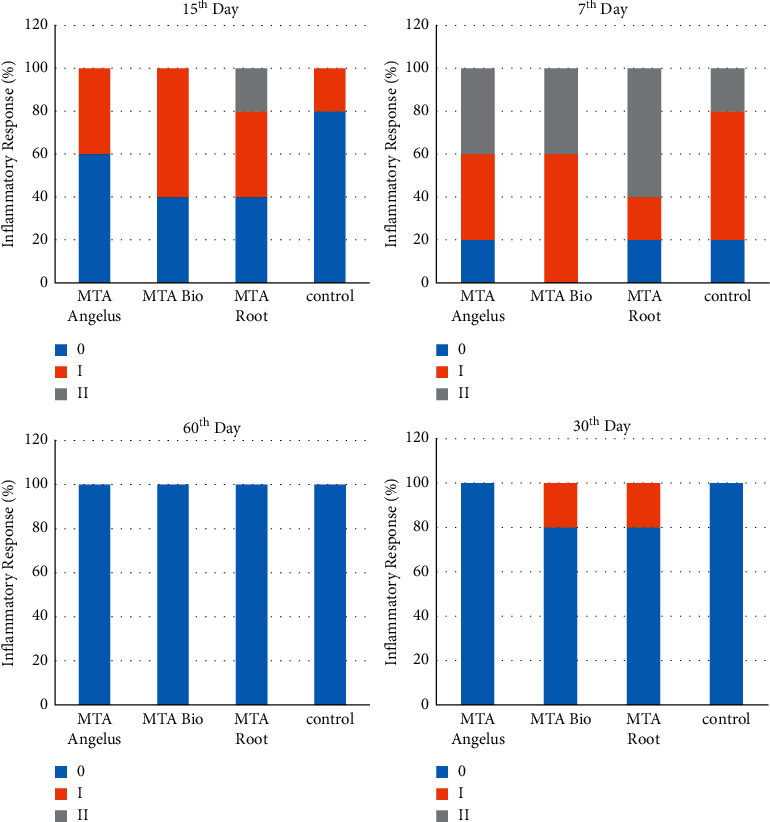
Mean values of inflammatory responses of different groups on days 7, 15, 30, and 60.

Histological findings are presented in Figure 1.  Figure 2 shows the mean values of inflammatory responses of different groups on days 7, 15, 30, and 60. There was no statistical difference between the tested materials and the mean scores of inflammation, cell gland, and granular tissue (*p* > 0.05).

The control group causes mild-to-moderate inflammatory reactions in subcutaneous connective tissues or no reaction. After 7 days, the Angelus MTA group showed a mild-to-moderate inflammatory response that decreased to a mild response after 15 days. The inflammatory response was suppressed after 30 and 60 days and showed no reaction. The BioMTA group showed a mild-to-moderate response after 7 days. The inflammatory response was lowered after 15 and 30 days, and ultimately, there was no response at 60 days. The Root MTA group showed mild-to-moderate inflammatory reactions on days 7 and 15 after surgery. This postoperative response was lowered to mild at 30 days and no response at 60 days. After 7, 15, 30, and 60 days, there was no statistically significant difference between the mean score of inflammation in all groups (*p* > 0.05).

**Figure 3:**
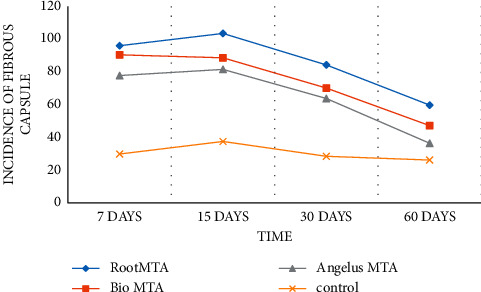
Fibrous capsule sizes in different groups in 7, 15, 30, and 60 days.

Fibrous capsule formation increased on 15 days in all groups except BioMTA. In the BioMTA, Angelus MTA, Root MTA, and the control group, the formation of fibrous capsules was decreased after 30 and 60 days. There was a statistically significant difference between the formation of fibrous capsules in different experimental periods (*p* < 0.05) (Figure 3 and Table 1).

Fibrous capsule formation after 7 days was similar in the BioMTA and Root MTA and was more than in the Angelus MTA and the control group (Figures 1(a) and 1(b)). There was a statistically significant difference between BioMTA, Angelus MTA, and control groups (*p* < 0.05). After 15 (Figure 1(c)) and 30 days in BioMTA and Angelus groups, fibrous capsule formation was similar, less than in Root MTA and more than in the control group. There was a statistically significant difference between BioMTA, Root MTA, and control groups (*p* < 0.05). Fibrous capsule formation was evaluated after 60 days from the highest to the lowest, Root MTA, BioMTA, Angelus MTA, and control groups, respectively (Figure 1(d)). There was a statistically significant difference between all groups (*p*< 0.05). According to histological evaluations, the areas of dystrophic calcification were similar at different times, and there was no statistically significant difference (*p* > 0.05). Granular tissue formation was similar in all groups after 7, 15, 30, and 60 days (*p* > 0.05).

## 4. Discussion

Biological evaluation of the potential risks of any new dental material is essential before clinical use [16]. Most studies have focused on the tissue response to endodontic materials, including the implantation of materials in the tissue and histological examination [17]. In previous studies, implantation of materials in connective tissue has been carried out alone or by placing them in silicone, polyethylene, or dentin tubes [18]. There was no significant difference between the inflammatory responses of Root MTA, BioMTA, Angelus MTA, and MTA control in each follow-up period in the present study. However, there is a statistically significant difference between fibrous capsule formation in 4 groups during 7, 15, 30, and 60 days. According to the statistical results, the inflammation has improved over time. Yaltirik et al. performed a study to evaluate subcutaneous connective tissue responses to ProRoot MTA and high-copper oral amalgam (Coltene). These materials were placed in polyethylene tubes and implanted in the dorsal connective tissue of Wistar albino rats, and tissue samples were collected and histologically examined 7, 15, 30, 60, and 90 days after implantation [19]. Similar to the present study results, the formation of a thin fibrosis capsule around MTA ProRoot-filled polyethylene implant tubes was confirmed 30 days after implantation. In our study, a thin fibrosis capsule was formed 30 days after implantation. Another notable finding was dystrophic calcification in connective tissue adjacent to MTA ProRoot [19]. Our study's method and sample were similar to the those in the study mentioned, but we used the Iranian sample ProRoot MTA, Root MTA. Iranian MTA is a substance that is composed of hydrophilic particles, and colloidal gel is obtained by hydrating the particles of this substance. It is composed of calcium silicate, bismuth oxide, calcium aluminate, calcium aluminoferrite, and calcium sulfate. Iranian MTA, like other dental cements, does not harden quickly, and the accuracy of mixing the material makes it easier to use [20]. After 30 days, dystrophic calcification formed around implant-filled Root MTA polyethylene tubes. We also examined the formation of dystrophic calcification in tubes filled with Angelus MTA and BioMTA. The present study was performed in 60 days, while Yaltirik et al. [19] examined connective tissue response to ProRoot MTA and high-copper oral amalgam over 90 days. Mutoh et al. reported that implantation of the materials using a sterile tube prevents their release into the peripheral tissue. These tubes are similar to the tooth root canal and are more effective than placing the material directly into the tissue. The ineffectiveness of polyethylene tubes makes them suitable for planting studies [21]. In the present study, a sterile polyethylene tube was used for implantation. Due to the high similarity of these tubes to the root canal, this study can bring us closer to the human body's real conditions. In another study, the biocompatibility of two modified Portland Cement (PC) formulations (PC combined with titanium oxide and PC combined with titanium oxide and calcium chloride) in rat subcutaneous tissue was investigated. Angelus MTA was considered as a control. The results of this study showed that the modified PCs have proper biocompatibility [15]. Angelus MTA is obtained by adding titanium oxide to MTA [22]. Titanium oxide has been introduced as a new material in dentistry due to its catalytic activity and biocompatibility [15]. It has also been reported that root canal sealers containing titanium oxide have antimicrobial components [23] and these materials can improve mechanical properties [24], including fracture resistance, compressive strength, flexural strength, and microtensile bond strength [25]. In this study, the antimicrobial properties of the materials were not examined, so it would be better to consider this in future studies. The formation of fibrous capsules indicates a high response of tissues to the materials. In this study, a thin, fibrous capsule without dense collagen was formed. The Root MTA group showed more fibrous capsules in all intervals than the other groups. There was also a significant difference between the formation of fibrous capsules in Root MTA, BioMTA, and Angelus MTA and MTA control groups. The formation of fibrous capsules in the Root MTA group in all periods is probably due to differences in the nature of this substance and reduces the biocompatibility of this substance. The mean thickness of fibrous capsules was used to evaluate the biocompatibility of the materials. In other studies, the formation of thick fibrous capsules around the implanted material has been observed due to their lower biocompatibility [26].

## 5. Conclusions

According to this study, these materials had good biocompatibility. Although the biocompatibility of all three MTAs used in this study was evaluated as acceptable, the inflammatory response of Angelus MTA was decreased more quickly and produced fewer fibrous capsules. So, it seems to have a better bioavailability than others.

## Figures and Tables

**Table 1:** Mean scores and standard deviation of the fibrous capsule after 7, 15, 30, and 60 days and *p* value for comparison of test groups and each group in different time periods.

Time	7 days	15 days	30 days	60 days
Groups	Mean ± SD			
Control	29/86 ± 3/36^a^	37/54 ± 2/90^a^	28/48 ± 3/66^a^	26/20 ± 3/39^a^
MTA Angelus	77/76 ± 6/62^b^	81/52 ± 7/21^b^	63/76 ± 5/27^b^	36/44 ± 4/46^b^
MTA Bio	90/40 ± 6/41^c^	88/58 ± 5/19^b^	70/14 ± 4/63^b^	47/20 ± 6/16^c^
MTA Root	95/96 ± 5/77^c^	103/46 ± 8/99^c^	84/26 ± 4/51^c^	59/72 ± 3/11^d^
*p* value	<0.001	<0.001	<0.001	<0.001

Different letters do not show statistical differences in each column.

## Data Availability

The data used to support the findings of this study are included within the article.
